# Supplementary material for: Zn2+-Dependent Nuclease Is Involved in Nuclear Degradation during the Programmed Cell Death of Secretory Cavity Formation in Citrus grandis ‘Tomentosa’ Fruits
Source: Cells. 2021 Nov 18;10(11):3222. doi: 10.3390/cells10113222 (PMC8622950; doi:10.3390/cells10113222)
Supplement: Supplementary file 1 [file cells-10-03222-s001.zip › cells-1436984-supplementary.pdf]

Table S1. The sequence of gene specific primers.

Gene	**Forward(5'-3')**	**Reserve(5'-3')**
CgENDO1(RT)	GAAGCTCTGATCGCCGACAT	GATTTGTGCCTGAACCAGCG
β-Actin	CACACTGGAGTGATGGTTGG	ATTGGCCTTGGGGTTAAGAG
CgENDO1(Clone)	ATGGGGGGATTGTGCCCTTTA	TCATGTAGCTGCTGCAAATTC TTC
CgENDO1(PGEX)	TGGATCCCCGGAATTCATGGG GGGATTGTGCCCTTTA	CTCGAGTCGACCCGGTCATGT AGCTGCTGCAAATTCTTC

Note: β-Actin is the internal control for quantitative real-time PCR. *CgENDO1(RT)* is for quantitative real-time PCR. *CgENDO1(Clone)* is for gene clone; *CgENDO1(PGEX)* is for expression of *CgENDO1* in the *in vitro* Escherichia coli.

A

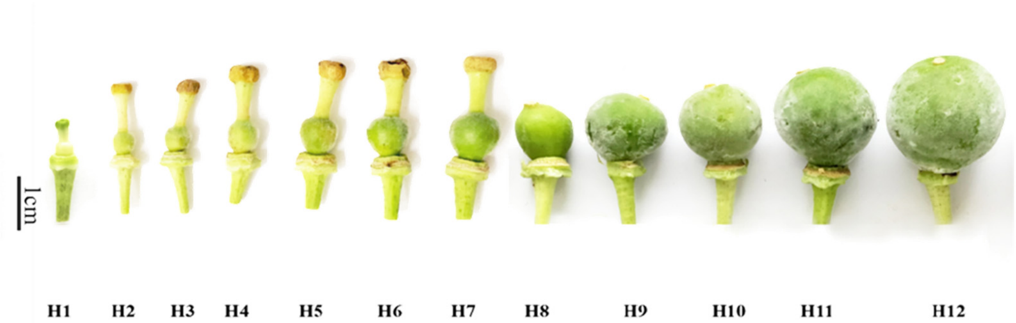

B

Stage	H1	H2	H3	H4	H5	H6	H7	H8	H9	H10	H11	H12
Size (length × width) (mm)	2×2	3×3	4×4	6×5	7×5	8×5	8×7	10×10	13×12	17×17	19×19	22×22

C

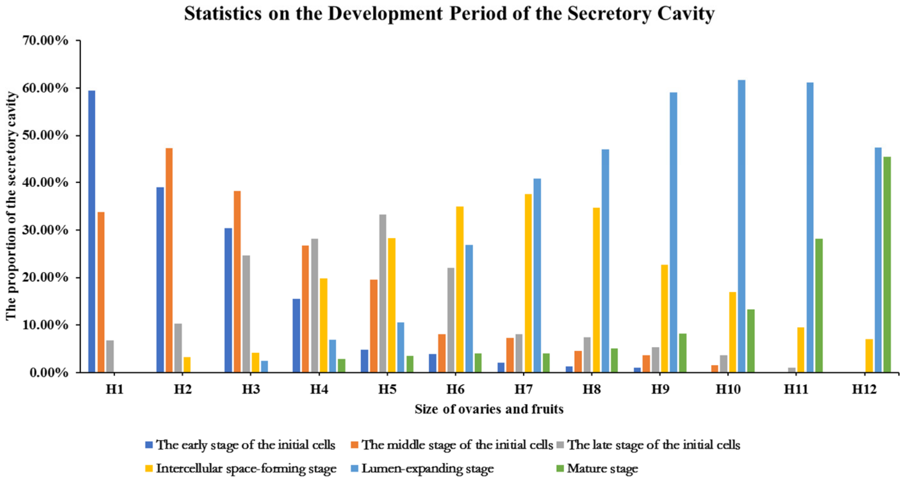

Figure S1. Statistics of sampling standards at different developmental stages of the secretory cavity of *Citrus grandis* 'Tomentosa' fruits.

A. Morphology of *Citrus grandis* 'Tomentosa' fruits.

B. Sampling standard of different developmental stages of secretory cavity of *Citrus grandis* 'Tomentosa' fruits.

C. Proportion statistics of secretory cavity development stages in different fruit sizes. Sample form: diameter (size, 1mm×1mm); Sample quantity: 20.

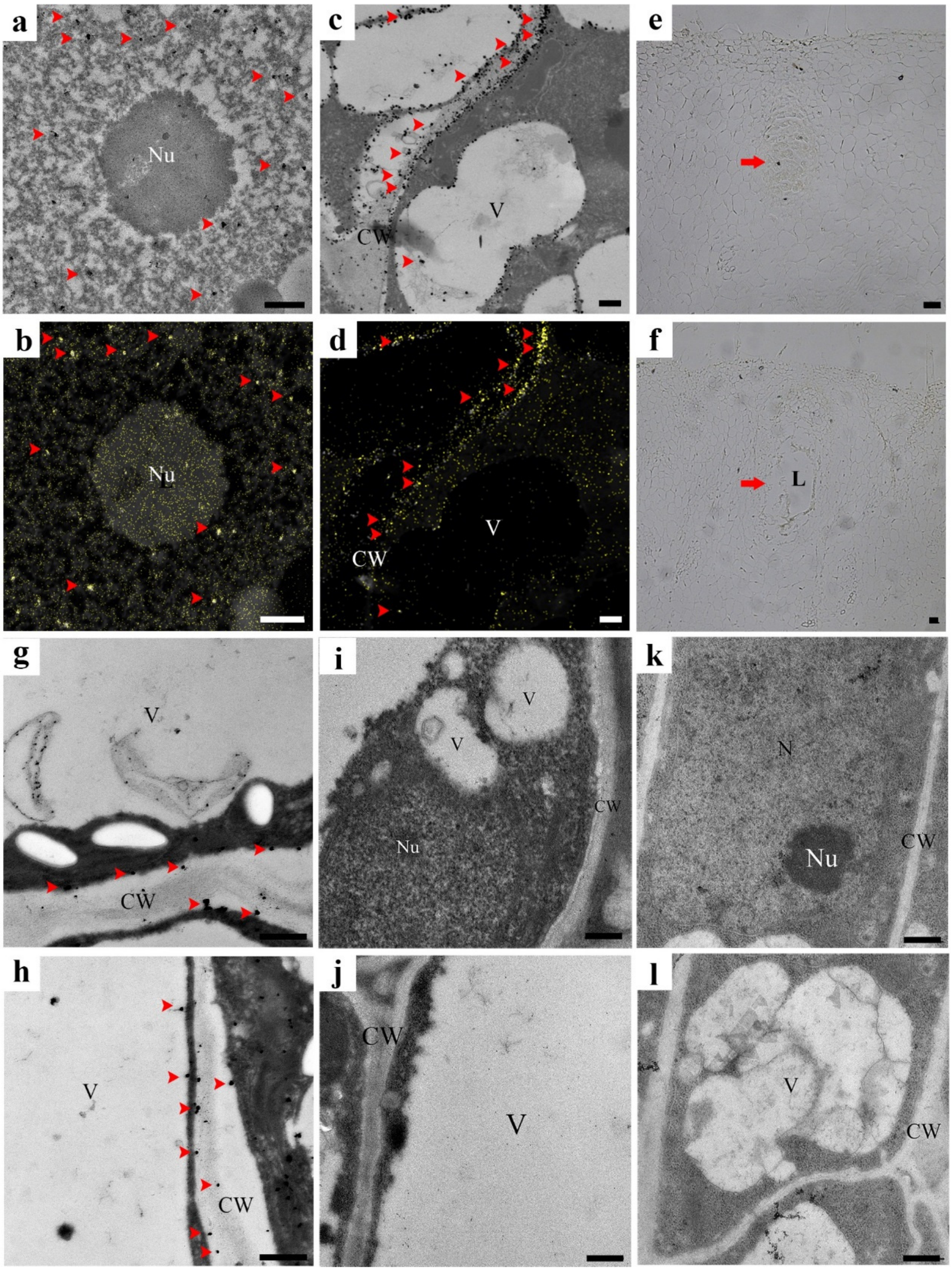

Figure S2. Control.

(a)-(d) Zn^{2+} subcellular localization methodology validation, which display EDS spectrum analysis of secretory cavity cells of *Citrus grandis* 'Tomentosa' fruits. (a) shows the black precipitations in the nucleus (arrowhead), (b) shows the black precipitations in the cell walls (arrowhead), (c) shows black precipitation in the nucleus

contains Ag elements (arrowhead), (d) shows black precipitation in the cell walls contains Ag elements (arrowhead).

(e)-(f) *in situ* hybridization control.

(g)-(h) Zn^{2+} subcellular localization control. (g), (h) shows non-secretory cavity cells of *Citrus grandis* 'Tomentosa' fruits. There are little Zn^{2+} in the cell wall (arrowhead).

(i)-(l) Immunogold particles control. (i), (j) shows non-secretory cavity cells. There are no immunogold particles in the nucleus, vacuole and cell wall. (k), (l) shows the globular regoin. (k) shows control in which the antibody replaced with pre-immune serum. (l) shows control which is used PBST instead of the antibody.

N: Nucleus; Nu: Nucleolus; V: Vacuole; CW: Cell Wall.

Bar = 500 nm.

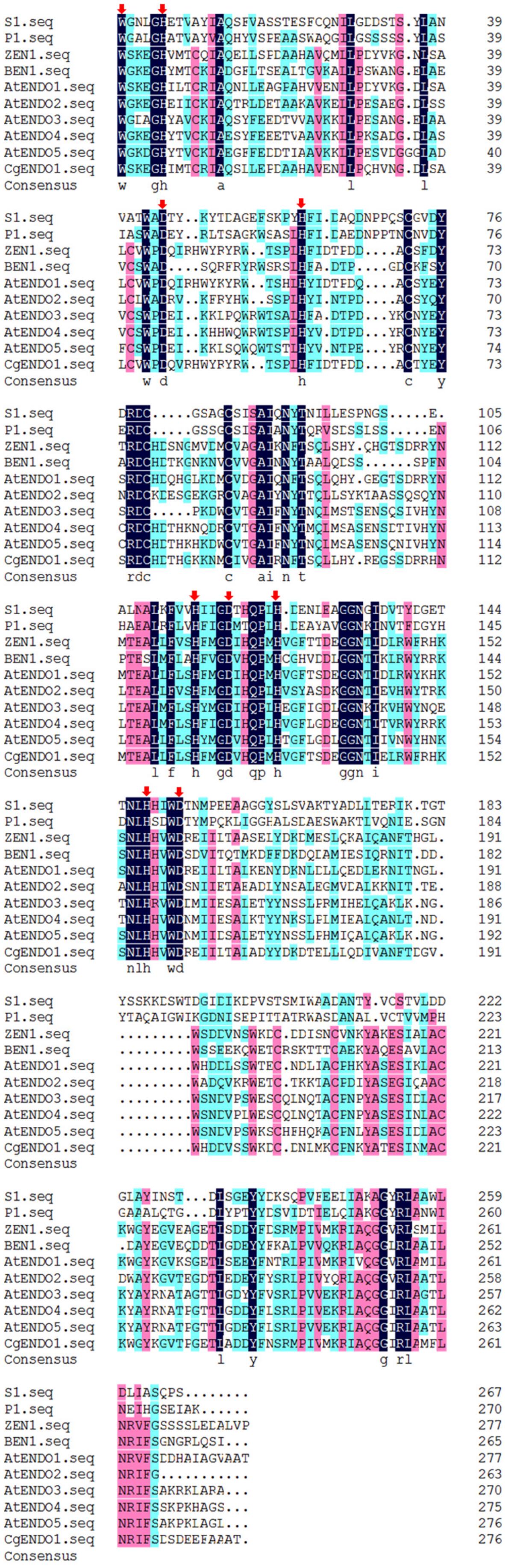
S1.seq WGNLGHETVAYIAQSFVASSTESFCQNILGDDSTS.YIAN 39
P1.seq WGALGHATVAYVAQHYVSPEAASWAQGILGSSSSS.YLAS 39
ZEN1.seq WSKEGHVMTCQIAQELLSPDAAHAVQMILPDYVKG.NLSA 39
BEN1.seq WGKEGHYMTCKIADGFLTSEALTGVKAILPSWANG.ELAE 39
AtENDO1.seq WSKEGHILTCRIAQNLLEAGPAHVVENILPDYVKG.DLSA 39
AtENDO2.seq WGKEGHEIICKIAQTRLDETAAKAVKEILPESAEG.DLSS 39
AtENDO3.seq WGLAGHYAVCKIAQSYFEEDTVVAVKKILPESANG.ELAA 39
AtENDO4.seq WGKEGHYTVCKIAESYFEEETVAAVKKILPKSADG.DLAS 39
AtENDO5.seq WGKDGHYTVCKIAEGFFEDDTIAAVKKILPESVDGGGLAD 40
CgENDO1.seq WSKEGHIMTCRIAQSILEPDAAHAVENILPQHVNG.DLSA 39
Consensus w gh a l l
S1.seq VATWADTY..KYTDAGEFSKPYHFI.DAQDNPPQSCGVDY 76
P1.seq IASWADEY..RLTSAGKWSASIHFI.DAEDNPPTNCNVDY 76
ZEN1.seq LCVWPDQIRHWYRYRW..TSPLHFIDTPDD....ACSFDY 73
BEN1.seq VCSWAD....SQRFRYRWSRSLHFA.DTP....GDCKFSY 70
AtENDO1.seq LCVWPDQIRHWYKYRW..TSHLHYIDTPDQ....ACSYEY 73
AtENDO2.seq LCIWADRV..KFRYHW..SSPLHYI.NTPD....ACSYQY 70
AtENDO3.seq VCSWPDEI..KKLPQWRWTSALHFA.DTPD...YKCNYEY 73
AtENDO4.seq VCSWPDEI..KHHWQWRWTSPLHYV.DTPD...YRCNYEY 73
AtENDO5.seq FCSWPDEI..KKLSQWQWTSTLHYV.NTPE...YRCNYEY 74
CgENDO1.seq LCVWPDQVRHWYRYRW..TSPLHFIDTPDD....ACTYEY 73
Consensus w d h c y
S1.seq DRDC.....GSAGCSISAIQNYTNILLESPNGS.....E. 105
P1.seq ERDC.....GSSGCSISAIANYTQRVSDSSISS.....EN 106
ZEN1.seq TRDCHDSNGMVDMCVAGAIKNFTSQLSHY.QHGTSDRRYN 112
BEN1.seq ARDCHDTKGNKNVCVVGAINNYTAALQDSS......SPFN 104
AtENDO1.seq SRDCHDQHGLKDMCVDGAIQNFTSQLQHY.GEGTSDRRYN 112
AtENDO2.seq NRDCKDESGEKGRCVAGAIYNYTTQLLSYKTAASSQSQYN 110
AtENDO3.seq SRDC.....PKDWCVTGAIFNYTNQIMSTSENSQSIVHYN 108
AtENDO4.seq CRDCHDTHKNQDRCVTGAIFNYTMQIMSASENSDTIVHYN 113
AtENDO5.seq CRDCHDTHKHKDWCVTGAIFNYTNQIMSASENSQNIVHYN 114
CgENDO1.seq SRDCHDTHGKKNMCIVGAIRNFTSQLLHY.REGSSDRRHN 112
Consensus rdc c ai n t
S1.seq ALNALKFVVHIIGDTHQPLH.DENLEAGGNGIDVTYDGET 144
P1.seq HAEALRFLVHFIGDMTQPLH.DEAYAVGGNKINVTFDGYH 145
ZEN1.seq MTEALLFVSHFMGDIHQPMHVGFTTDEGGNTIDLRWFRHK 152
BEN1.seq PTESIMFLAHFVGDVHQPMHCGHVDDLGGNTIKLRWYRRK 144
AtENDO1.seq MTEALLFLSHFMGDIHQPMHVGFTSDEGGNTIDLRWYKHK 152
AtENDO2.seq LTEALLFVSHFMGDIHQPLHVSYASDKGGNTIEVHWYTRK 150
AtENDO3.seq LTEAIMFLSHYMGDIHQPLHEGFIGDLGGNKIKVHWYNQE 148
AtENDO4.seq LTEAIMFLSHFIGDIHQPLHVGFLGDEGGNTITVRWYRRK 153
AtENDO5.seq LTEALLFLSHYMGDVHQPLHTGFLGDLGGNTIIVNWYHNK 154
CgENDO1.seq MTEALLFLSHFMGDVHQPMHVGFTSDEGGNTIELRWFRHK 152
Consensus l f h gd qp h ggn i
S1.seq TNIHHIWDTNMPEEAAGGYSLSVAKTYADLITERIK.TGT 183
P1.seq DNIHSDWDTYMPQKLIGGHALSDAESWAKTIVQNIE.SGN 184
ZEN1.seq SNIHHVWDREIILTAASELYDKDMESLQKAIQANFTHGL. 191
BEN1.seq SNIHHVWDSDVITQTMKDFFDKDQDAMIESIQRNIT.DD. 182
AtENDO1.seq SNIHHVWDREIILTALKENYDKNLDLLQEDLEKNITNGL. 191
AtENDO2.seq ANIHHIWDSNIIETAEADLYNSALEGMVDAIKKNIT.TE. 188
AtENDO3.seq TNIHRVWDIMIIESALETYYNSSLPRMIHELQAKLK.NG. 186
AtENDO4.seq TNIHHVWDNMIIESALKTYYNKSLPLMIEALQANLT.ND. 191
AtENDO5.seq SNIHHVWDNMIIDSALETYYNSSLPHMIQALQAKLK.NG. 192
CgENDO1.seq SNIHHVWDREIILTAIADYYDKDTELLIQDIVANFTDGV. 191
Consensus nlh wd
S1.seq YSSKKDSWTDGIDIKDPVSTSMIWAADANTY.VCSTVLDD 222
P1.seq YTAQAIGWIKGDNISEPITTATRWASDANAL.VCTVVMPH 223
ZEN1.seq ....WSDDVNSWKDC.DDISNCVNKYAKESIALAC 221
BEN1.seq .........WSSEEKQWETCRSKTTTCAEKYAQESAVLAC 213
AtENDO1.seq ....WHDDLSSWTEC.NDLIACPHKYASESIKLAC 221
AtENDO2.seq ....WADQVKRWETC.TKKTACPDIYASEGIQAAC 218
AtENDO3.seq .........WSNDVPSWESCQLNQTACPNPYASESIDLAC 217
AtENDO4.seq .........WSNDVPLWESCQLNQTACPNPYASESINLAC 222
AtENDO5.seq .........WSNDVPSWKSCHFHQKACPNLYASESIDLAC 223
CgENDO1.seq ....WHDDVSSWKDC.DNIMKCPNKYATESINMAC 221
Consensus
S1.seq GLAYINST...DLSGEYYDKSQPVFEELIAKAGYRLAAWL 259
P1.seq GAAALQTG...DLYPTYYDSVIDTIELQIAKGGYRLANWI 260
ZEN1.seq KWGYEGVEAGETLSDDYFDSRMPIVMKRIAQGGVRLSMIL 261
BEN1.seq .DAYEGVEQDDTLGDEYYFKALPVVQKRLAQGGLRLAAIL 252
AtENDO1.seq KWGYKGVKSGETLSEEYFNTRLPIVMKRIVQGGVRLAMIL 261
AtENDO2.seq DWAYKGVTEGDTLEDEYFYSRLPIVYQRLAQGGVRLAATL 258
AtENDO3.seq KYAYRNATAGTTLGDYYFVSRLPVVEKRLAQGGIRLAGTL 257
AtENDO4.seq KYAYRNATPGTTLGDDYFLSRLPIVEKRLAQGGIRLAATL 262
AtENDO5.seq KYAYRNATPGTTLGDEYFLSRLPVVEKRLAQGGIRLAATL 263
CgENDO1.seq KWGYKGVTPGETLADDYFNSRMPIVMKRIAQGGIRLAMFL 261
Consensus l y g rl
S1.seq DLIASQPS........ 267
P1.seq NEIHGSEIAK...... 270
ZEN1.seq NRVFGSSSSLEDALVP 277
BEN1.seq NRIFSGNGRLQSI... 265
AtENDO1.seq NRVFSDDHAIAGVAAT 277
AtENDO2.seq NRIFG........... 263
AtENDO3.seq NRIFSAKRKLARA... 270
AtENDO4.seq NRIFSSKPKHAGS... 275
AtENDO5.seq NRIFSAKPKLAGL... 276
CgENDO1.seq NRIFSDSDEEFAAAT. 276
Consensus

Figure S3. Comparison of the functional domains of CgENDO1 and different Zn^{2+} dependent nucleases and zinc ion binding sites involved in PCD.

S1 = *Aspergillus oryzae* 3.042 (EIT80979.1); P1 = *Penicillium citrinum* (P24289.1); ZEN1 = *Zinnia elegans* (BAA28948.1); BEN1 = *Hordeum vulgare* subsp. Vulgare (BAA28942.1); AtENDO1 (BFN1) = *Arabidopsis thaliana* (AAD00693.1); AtENDO2 = *Arabidopsis thaliana (*NP_176996.1); AtENDO3 = *Arabidopsis thaliana* (OAO99578.1); AtENDO4 = *Arabidopsis thaliana* (NP_001328107.1); AtENDO5 = *Arabidopsis thaliana* (OAO96613.1) (Arrow indicates conserved sequence to bind three Zn^{2+} when enzymes work.)
